# FTIR-Derived Feature Insights for Predicting Time-Dependent Antibiotic Resistance Progression

**DOI:** 10.3390/antibiotics14080831

**Published:** 2025-08-15

**Authors:** Mitchell Bonner, Claudia P. Barrera Patiño, Andrew Ramos Borsatto, Jennifer M. Soares, Kate C. Blanco, Vanderlei S. Bagnato

**Affiliations:** 1 Biomedical Engineering, Texas A&M University, 400 Bizzell St, College Station, TX 77843, USA; andrewrborsatto@tamu.edu (A.R.B.); jennifer.soares@usp.br (J.M.S.); or bagnatovs@tamu.edu (V.S.B.); 2 Sao Carlos Institute of Physics, University of Sao Paulo, IFSC-USP, Sao Carlos 13566-590, SP, Brazil; kateblanco@ifsc.usp.br

**Keywords:** antibiotic-resistant bacteria, prediction model, *Staphylococcus aureus*

## Abstract

Background/Objectives: The progression of antibiotic resistance is increasingly recognized as a dynamic and time-dependent phenomenon, challenging conventional diagnostics that define resistance as a binary trait. Methods: Biomolecules have fingerprints in Fourier-transform infrared spectroscopy (FTIR). The targeting of specific molecular groups, combined with principal component analysis (PCA) and machine learning algorithms (ML), enables the identification of bacteria resistant to antibiotics. Results: In this work, we investigate how effective classification depends on the use of different numbers of principal components, spectral regions, and defined resistance thresholds. Additionally, we explore how the time-dependent behavior of certain spectral regions (different biomolecules) may demonstrate behaviors that, independently, do not capture a complete picture of resistance development. FTIR spectra were obtained from *Staphylococcus aureus* exposed to azithromycin, trimethoprim/sulfamethoxazole, and oxacillin at sequential time points during resistance induction. Combining spectral windows substantially improved model performance, with accuracy reaching up to 96%, depending on the antibiotic and number of components. Early resistance patterns were detected as soon as 24 h post-exposure, and the inclusion of all three biochemical windows outperformed single-window models. Each spectral region contributed distinctively, reflecting biochemical remodeling associated with specific resistance mechanisms. Conclusions: These results indicate that antibiotic resistance should be viewed as a temporally adaptive trajectory rather than a static state. FTIR-based biochemical profiling, when integrated with ML, enables projection of phenotypic transitions and supports real-time therapeutic decision-making. This strategy represents a shift toward adaptive antimicrobial management, with the potential to personalize interventions based on dynamic resistance monitoring through spectral biomarkers.

## 1. Introduction

Recently, there have been reported cases of resistance to nearly every antibiotic we have available [1,2], suggesting an increase in clinical cases of antibiotic-resistant infections worldwide. There is a dire need to find better treatment strategies using existing antibiotics that can slow or even reverse the development of resistance [2]. In the same vein, new strategies for detecting resistance quickly and accurately are needed. This demand includes not only faster diagnostics but also approaches capable of dynamically tracking the progression of bacterial adaptation to antimicrobials. Resistance should no longer be interpreted as a binary event but as a temporally structured and adaptive trajectory.

It was recently demonstrated by Barrera et al. that the use of FTIR, when directed at specific biochemical groups and coupled with PCA and machine learning algorithms, can successfully detect resistant bacterial phenotypes and identify antibiotic susceptibility [3,4]. These works confirmed that FTIR (Fourier-transform infrared spectroscopy) spectra of bacterial samples can be used to identify time-dependent antibiotic resistance development [3,4]. FTIR is an analytical technique that utilizes the absorption of infrared radiation to characterize the profiles of biomolecules in bacterial cells, which can help differentiate between different bacteria and develop analyses of samples with and without antibiotic resistance [3]. Such analyses consider that different bacterial species have distinct molecular compositions resulting in unique FTIR spectra for each species, including bacterial strains. These features were detected and identified through the implementation of machine learning algorithms used with different windows inside FTIR spectra from samples of *Staphylococcus aureus*, *Streptococcus pyogenes*, *Streptococcus mutans*, *Escherichia coli*, and *Klebsiella pneumoniae* [3,4]. Each biochemical spectral window such as carbohydrates, fatty acids, and proteins captures distinct aspects of cellular adaptation. Their combined analysis enhances classification accuracy by integrating complementary structural information.

Previous work has demonstrated the importance of utilizing machine learning algorithms in research aimed at identifying antibiotic resistance [3,4]. A methodology has been established to analyze the FTIR structural profiles of samples from Gram-positive and Gram-negative bacteria, and it was tested on hidden samples from clinical sources [3,4]. These prior works, which demonstrate cross-sectional applicability, focus on different microorganisms and reinforce the premise that the structural characteristics identified are universal among the microorganisms tested. Such models are not only useful for classification but can also be trained to detect early-stage resistance phenotypes, even before complete resistance is phenotypically expressed, allowing for earlier clinical intervention.

Changes in bacterial structures, metabolic alterations resulting from external stimuli such as the presence of antimicrobials, and genetic mutations or gene acquisition through conjugation can all lead to the development of resistance [5,6]. Resistance to antibiotics is an evolutionary response of bacteria to withstand and survive the effects of a stressor [1]. In this way, experiments using minimum inhibitory concentration (MIC) time-dependence data can be developed to investigate antibiotic resistance in bacterial pathogens, yielding information for the design of strategies to improve infection treatment [1,2,7,8]. This is the motivation behind this paper. Understanding how resistance emerges across temporal scales is essential for guiding the duration, intensity, and alternation of antibiotic therapies.

In previous work, the time-dependent behavior of resistance evolution in *S. aureus* (NIST 0023) was observed for three different antibiotics: azithromycin (Azy), trimethoprim/sulfamethoxazole (Trim), and oxacillin (Oxa) [9]. It was determined that the minimum inhibitory concentration (MIC) curves begin at low values and increase with time up to 120 h. In samples of *S. aureus* exposed to various concentrations of antibiotics, the MIC curves indicate a sustained increase for Azy and Trim, and a sudden decrease for Oxa after approximately 72 h [9].

The importance of time in determining bacterial resistance to antibiotics is demonstrated in this study of *S. aureus* samples collected at varying time points during resistance development. This work integrates microbiological techniques used to prepare the samples with machine learning algorithms applied to analyze a large quantity of FTIR spectral data. Principal component analysis (PCA) in multiple dimensions coupled with the use of many biochemical windows (each exhibiting unique average spectral behaviors) significantly improved the accurate classification of FTIR spectra from over one thousand samples. These included one hundred spectra per time point and per antibiotic, along with one shared control group. Results also demonstrated variation in resistance detection efficacy depending on defined resistance criteria and antibiotic exposure times. The capability to distinguish between non-exposed and early-stage-exposed samples as early as 24 h highlights the diagnostic value of this approach in clinical contexts, particularly for optimizing therapeutic windows.

The implemented methodology supports the goal of developing a safe, fast, and accurate way to detect bacterial susceptibility to antibiotics. The performance of a predictive model was evaluated to assess how feature selection affected this identification of resistance over time for three different antibiotics, and the results provide valuable insight into the relationship between antibiotic type, exposure time, and specific biomolecules. Notably, this methodology can be safely extended to other microorganisms and antibiotics. By enabling detection along the resistance timeline, it offers a foundation for more adaptable infection treatment. This points toward a paradigm shift in treatment—from reactive to anticipatory strategies.

## 2. Results

**Figure 1:**
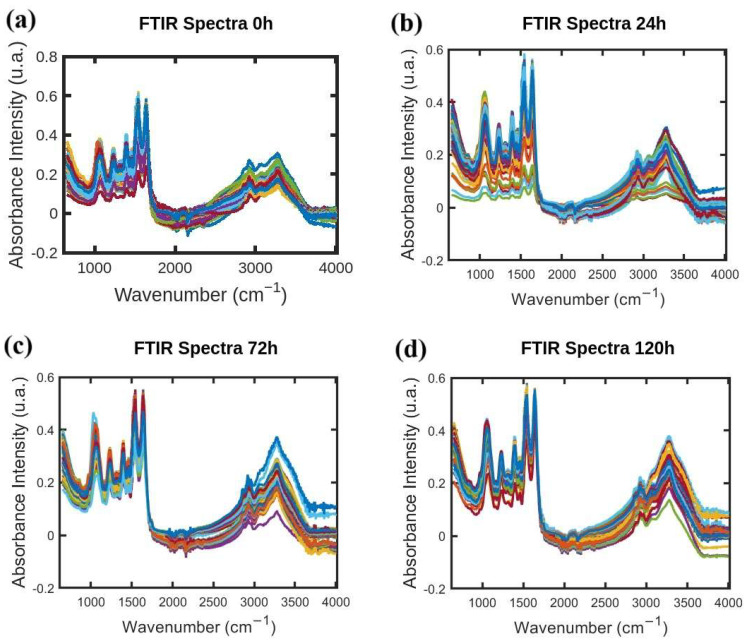
(**a**–**d**) FTIR absorption spectra of *S. aureus* samples collected at 0 h, 24 h, 72 h, and 120 h during resistance development for antibiotic Azy. Initial FTIR spectra data for vary times studied here has been employed in our previous work [9], with different kind of analyses.

**Figure 2:**
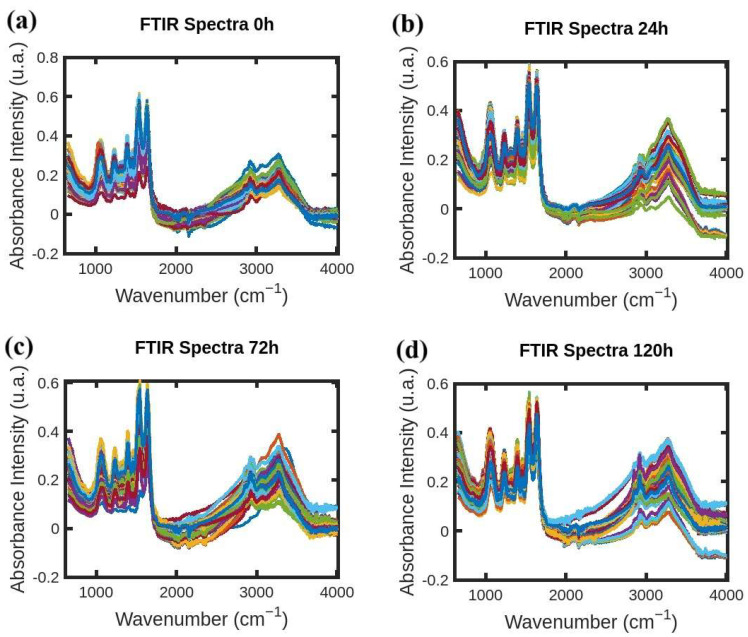
(**a**–**d**) FTIR absorption spectra of *S. aureus* samples collected at 0 h, 24 h, 72 h, and 120 h during resistance development for antibiotic Oxa.

**Figure 3:**
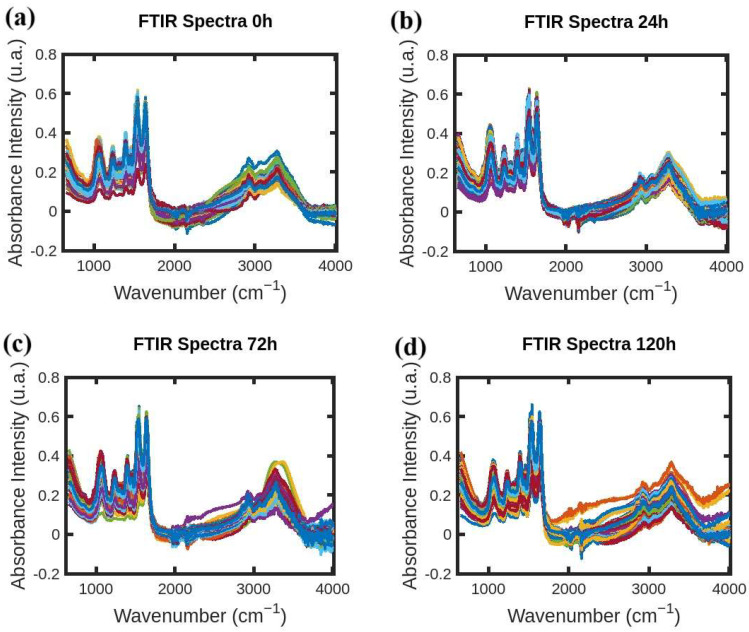
(**a**–**d**) FTIR absorption spectra of *S. aureus* samples collected at 0 h, 24 h, 72 h, and 120 h during resistance development for antibiotic Trim.

FTIR absorption spectra of *S. aureus* were acquired following the procedure developed by Soares et al. in [10,11] and the resistance-induced strain protocol reported in [12] by Soares et al. FTIR spectral data-processing code was developed by the authors in MATLAB (R2021b) [13] following the protocol reported by Naumann et al. [14]. One thousand FTIR absorption spectra of *S. aureus* were acquired in the wavelength range of 650–4000 cm^−1^. Three hundred FTIR spectra were acquired for each antibiotic with various exposure times of 24, 72, and 120 h (one hundred spectra each). Additionally, one hundred FTIR spectra were acquired for the “0 h” group with no exposure, and they accompany the sample sets unique to each antibiotic. Figure 1, Figure 2 and Figure 3 show FTIR spectra of *S. aureus* samples collected at various times during resistance development for three different antibiotics: azithromycin (Azy), oxacillin (Oxa), and trimethoprim/sulfamethoxazole (Trim).

The initial spectral data was first processed using second derivative analysis, smoothing, and min–max normalization before key biochemical regions for carbohydrates (950–1200 cm^−1^), proteins (1500–1800 cm^−1^), and fatty acids (2800–3100 cm^−1^) were isolated. PCA was applied within each antibiotic dataset to reduce dimensionality for better visualization and more focused model training. For classification, Random Forest models were used to test how well the development, or progression, of resistance could be detected across many antibiotic exposure times. The performance of these models was analyzed for each antibiotic utilizing two methods to study different aspects of the data and model. It was first evaluated by looking at the overall accuracy of trying to predict the time group (0 h, 24 h, 72 h, and 120 h) of each sample while considering both individual and combined biochemical windows. F1 scores were then analyzed for a binary classification of either “resistant” vs. “non-resistant” with difference criteria for what defined “resistant.” These findings bolster the paradigm shift in treatment outlined in the introduction, as the goal of understanding and predicting resistance development utilizing FTIR and machine learning is better realized.

The FTIR spectral profile peaks and the FTIR spectra normalized with the maximum values at 0, 24, 72, and 120 h for each biochemical window for the antibiotics Azy, Oxa, and Trim are shown in Figures S1–S9 in the Supplementary Materials. The results obtained from this process comprise the data used for the study of spectral regions utilizing PCA [3,4,9].

**Figure 4:**
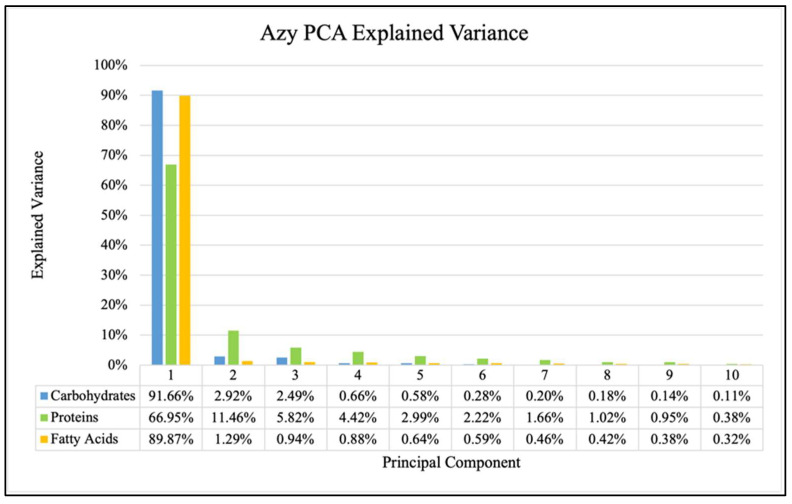
Explained variance for PCA of each biomolecule for Azy samples.

**Figure 5:**
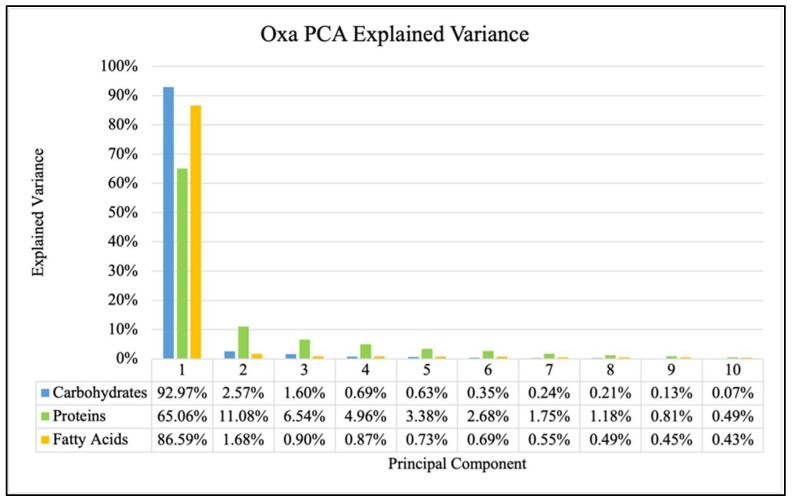
Explained variance for PCA of each biomolecule for Oxa samples.

**Figure 6:**
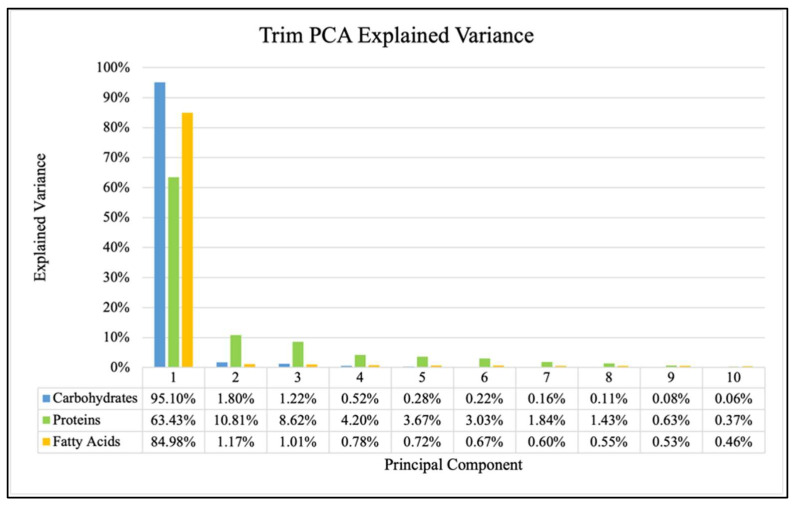
Explained variance for PCA of each biomolecule for Trim samples.

The explained variance (percentage of variance explained by each component) in each of the first ten principal components for all three antibiotics are shown in Figure 4, Figure 5 and Figure 6 and showed consistent trends. The amount of explained variance in the principal components for each biomolecule was found to be very high in the first component with a sharp drop in the second component followed by a steady decline. The protein windows always contained the lowest amount of explained variance in the first component, such that subsequent components explained more than those of the fatty acid and carbohydrate windows.

**Figure 7:**
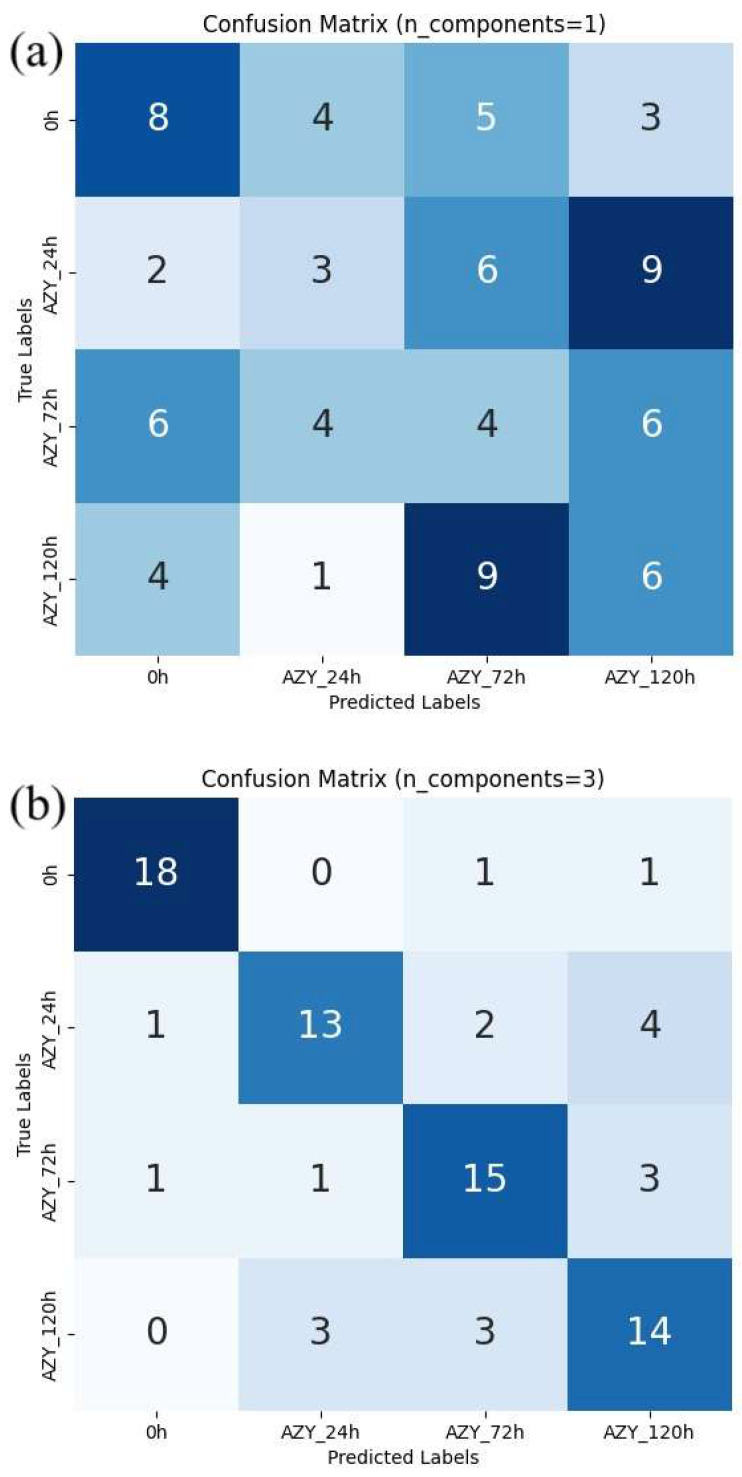
Confusion matrix examples for Azy sample classification. All windows included (**a**) oOne principal component per window; (**b**) three principal components per window.

**Figure 8:**
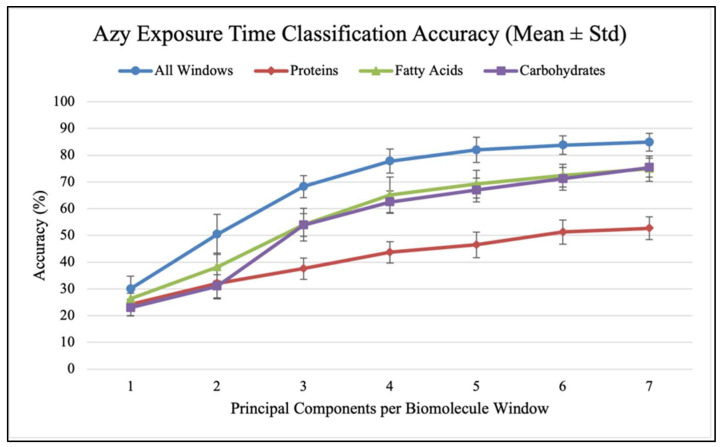
Classification accuracy vs. PC count for different biomolecules (Azy).

**Figure 9:**
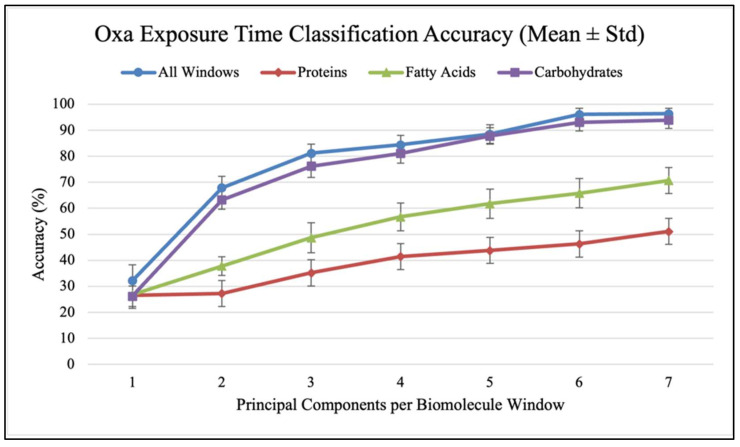
Classification accuracy vs. PC count for different biomolecules (Oxa).

**Figure 10:**
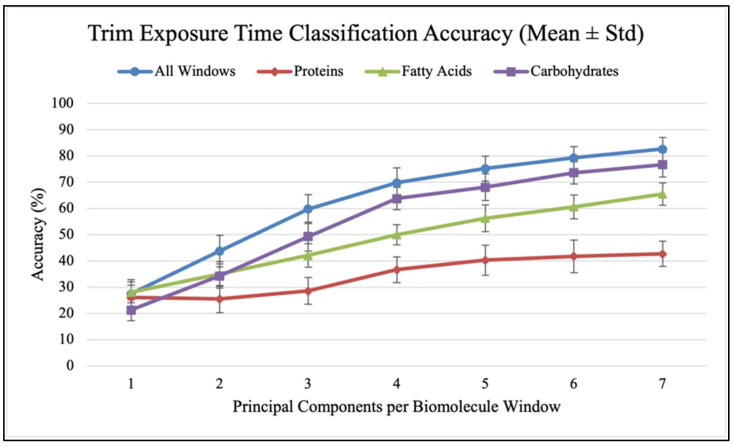
Classification accuracy vs. PC count for different biomolecules (Trim).

**Figure 11:**
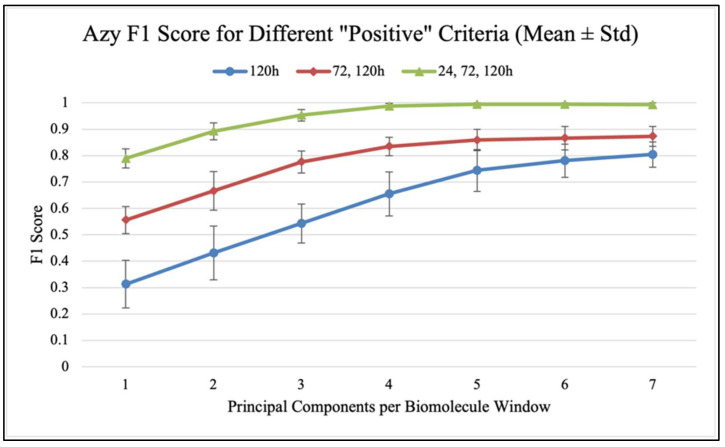
F1 score vs. PC count for different “positive” criteria (Azy).

**Figure 12:**
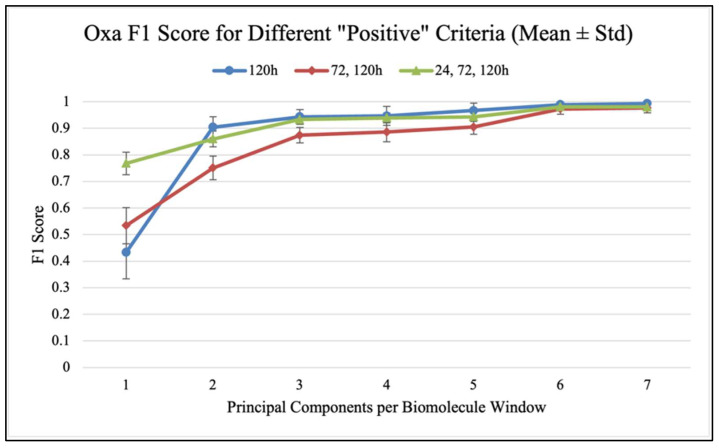
F1 score vs. PC count for different “positive” criteria (Oxa).

**Figure 13:**
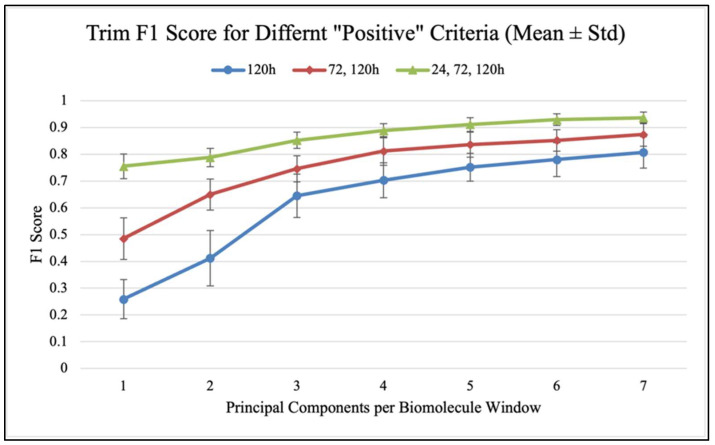
F1 score vs. PC count for different “positive” criteria (Trim).

Confusion matrices were used to evaluate the performance of classification models and calculate classification accuracy and F1 scores in different analysis scenarios. As shown in Figure 7, confusion matrices display the results used to calculate classification accuracy values and F1 scores. For each antibiotic, Figure 8, Figure 9 and Figure 10 show the overall classification accuracy when the principal components for each biochemical window are used as features as well as when all three groups of features are used together. Figure 11, Figure 12 and Figure 13 then show, using principal components from all three biochemical groups together, F1 scores for a binary classification using the three different criteria defining what is considered “positive” for resistance.

**Figure 14:**
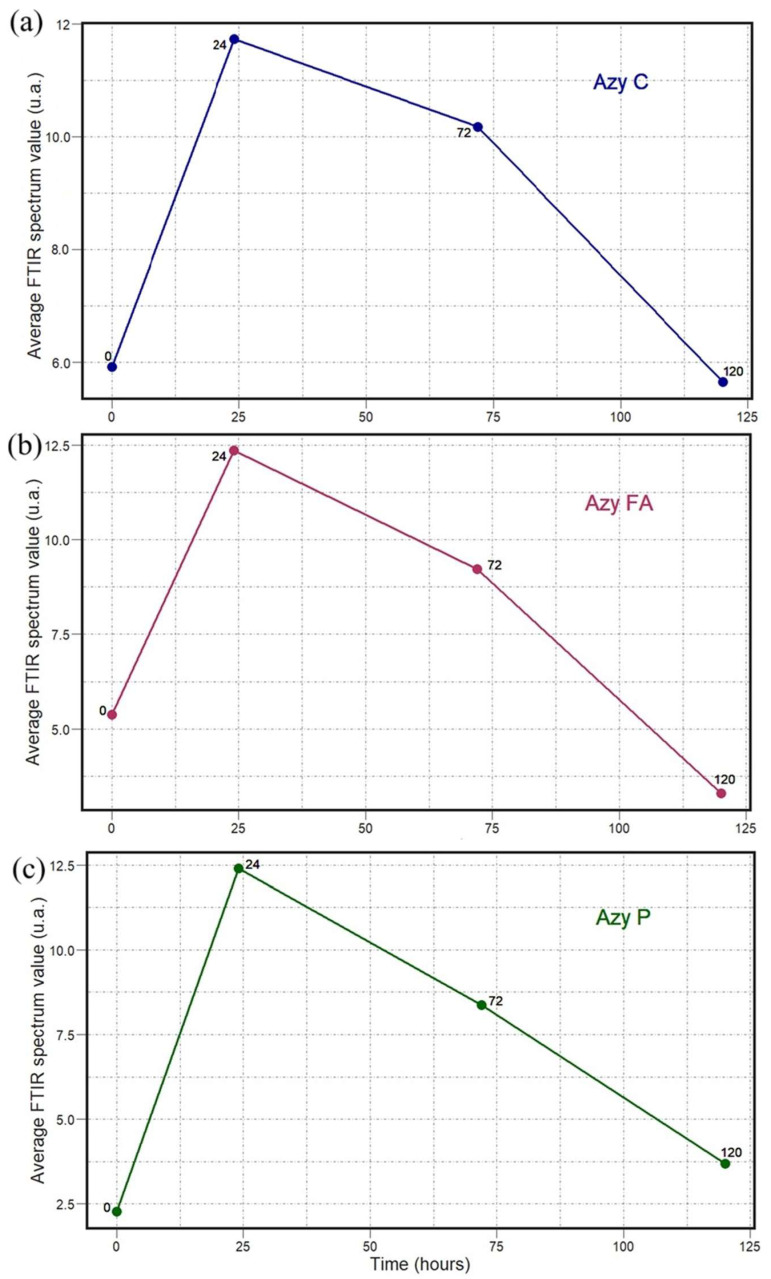
Temporal dependence of average FTIR spectra for *S. aureus* treated with Azy for (**a**) carbohydrate (C), (**b**) fatty acid (FA), and (**c**) protein (P) biochemical windows.

**Figure 15:**
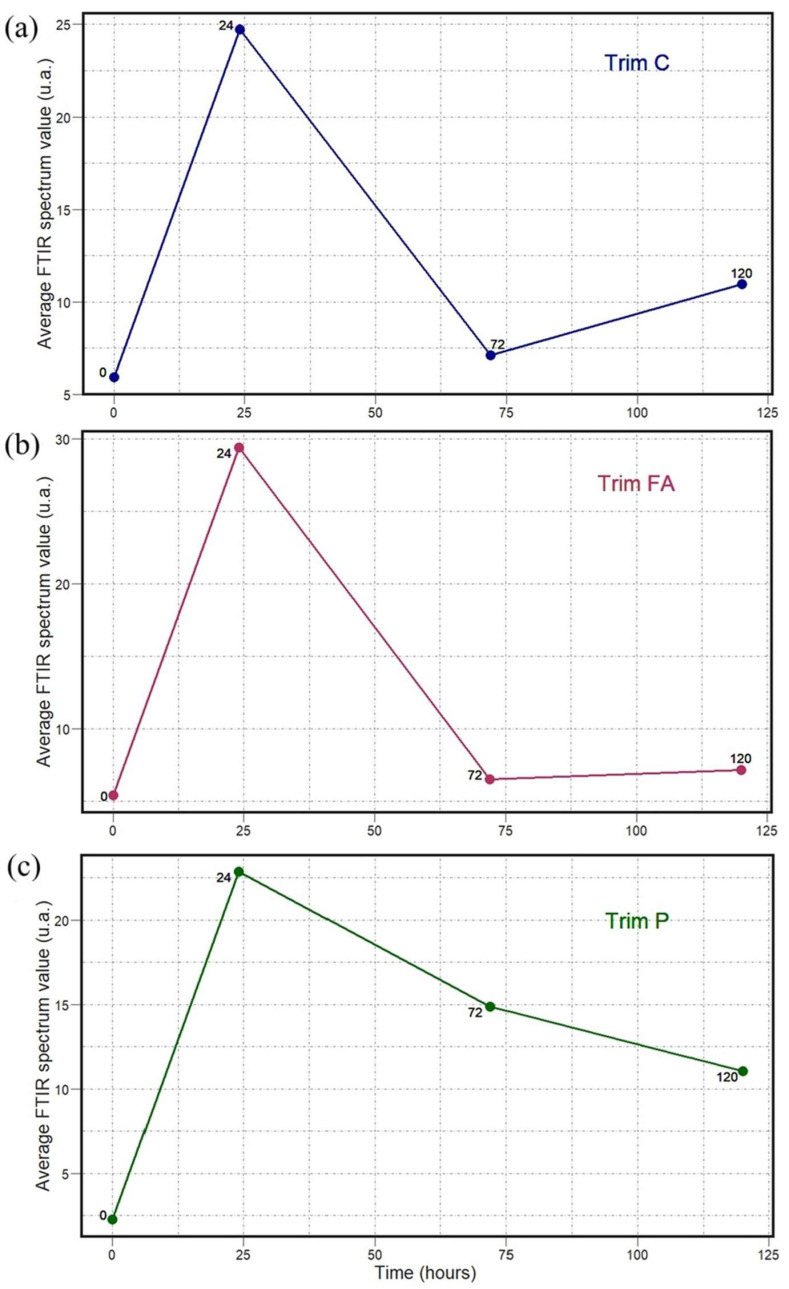
Temporal dependence of average FTIR spectra for *S. aureus* treated with Oxa for (**a**) carbohydrate (C), (**b**) fatty acid (FA), and (**c**) protein (P) biochemical windows.

**Figure 16:**
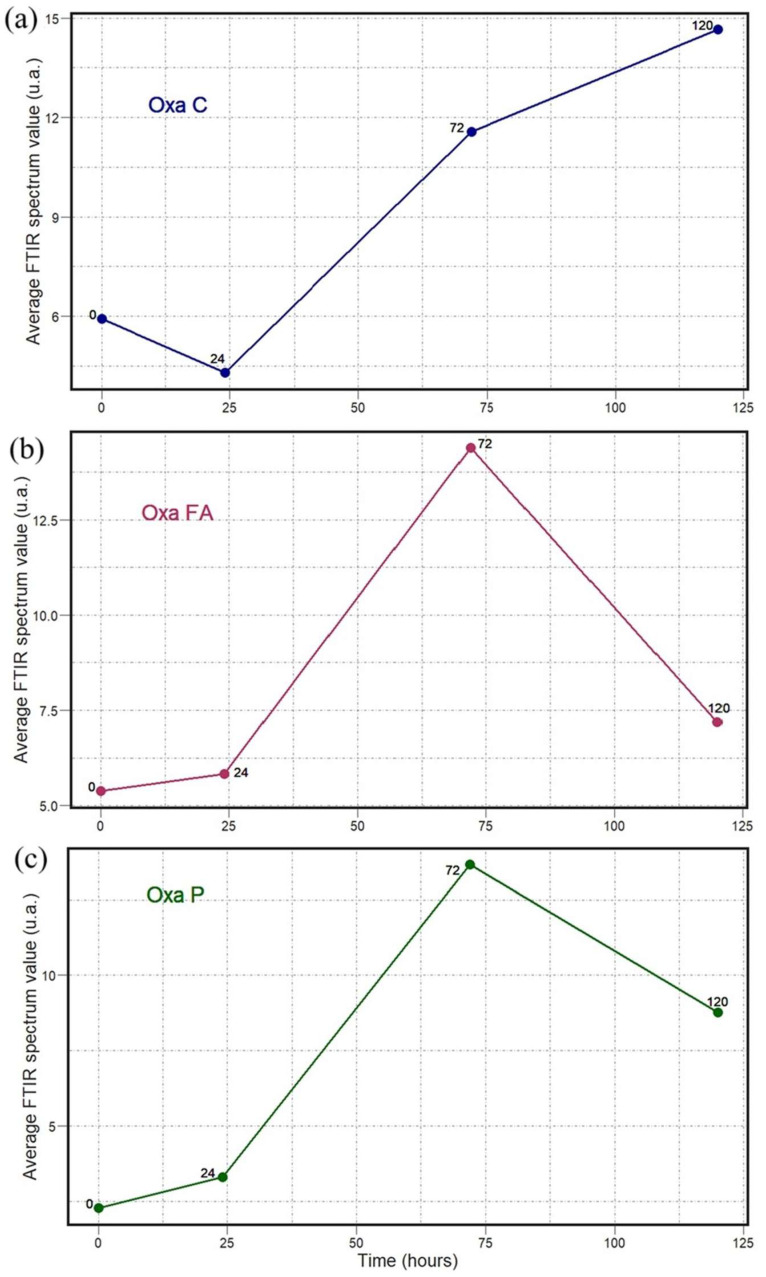
Temporal dependence of average FTIR spectra for *S. aureus* treated with Trim for (**a**) carbohydrate (C), (**b**) fatty acid (FA), and (**c**) protein (P) biochemical windows.

The results shown in Figure 8, Figure 9, Figure 10, Figure 11, Figure 12 and Figure 13 allow us to identify the general behavior of all FTIR spectral samples as it pertains to specific model application. All FTIR data were considered in the study of time evolution with methods that analyze the use of multiple principal components and the contribution of the different biochemical windows. Additionally, one more calculation was introduced to aid the interpretation of these results. The average FTIR spectrum was found for each biochemical window for carbohydrates, fatty acids, and proteins. This was carried out for each antibiotic and exposure time to include all samples of *S. aureus* studied. The results obtained are shown in Figure 14, Figure 15 and Figure 16.

## 3. Discussion

It is observed for each of the three analyzed biomolecules that the explained variance of the principal components drops sharply after the first one, though there are still significant amounts explained by subsequent components. This decrease from the first to the second component is consistently smallest in the protein window. This general behavior is true for all three antibiotics and can be observed in Figure 4, Figure 5 and Figure 6. This has been previously investigated and remains a critical consideration in identifying effective FTIR-derived features for antibiotic resistance detection models [9]. The calculations associated with all model testing were derived from confusion matrices like those shown in Figure 7. It is these classifications that enable the analysis of different performance metrics alongside the application of different criteria for what is considered “positive for resistance”.

Figure 8, Figure 9 and Figure 10 show how accuracy changes with the use of additional principal components for each individual window and then for all the window features used together for training. Metrics are only found up to seven components because this is where metrics generally plateau. Furthermore, each additional component introduces more noise, such that using this many components is likely increasing the model’s dependence on spectral patterns unique to the collection environment rather than the actual chemical changes that accompany resistance development. For all three antibiotics, the use of all three windows together produces higher accuracy than any of them individually. This indicates that each window provides unique data for each sample so that more patterns can be found across the time groups when used together. The shape of the data reveals two additional patterns. Firstly, it is the carbohydrate data that seems to consistently produce a higher accuracy among the individual groups and follows the grouped data curve the closest. This indicates that the data from the carbohydrate window is a significant contributor to the high accuracy when all windows are used together. This is perhaps contrary to what might be expected, as the protein window had the largest spread of explained variance across principal components. The second significant result is that the largest accuracy gains occur with the addition of the first few (two to four) components. Subsequent components increase performance, but with diminishing gains.

There are many methods for selecting the appropriate number of principal components to use in an analysis. Kaiser’s criterion, which includes only components with an eigenvalue greater than 1, represents an incredibly common method that is widely criticized for often retaining too many components, or unhelpful noise [15,16]. In our case, this criterion would mean keeping 3–5 carbohydrate components, 5–6 fatty acid components, and 9 protein components depending on the antibiotic. More complex methods for making component retention decisions should be applied, but this would need to be accompanied by classification testing with additional data collected in new environments. This simple method, therefore, serves the purpose of considering the results of this baseline exploration and likely providing the maximum number of components that could be reasonably retained for each window. In general, it is significant to note that this brief analysis of principal component contribution alongside a look at possible strategies for retention indicates that utilizing up to three or four principal components can lead to significant classification accuracy improvements that should be verified with new data. Table A1, Table A2 and Table A3 (In Appendix A) contain the values from these plots of overall classification accuracy.

The prior discussion is significant as it pertains to potential feature selection improvements, but this overall time-group classification is more complex than the actual goal of classifying samples as either resistant (positive) or non-resistant (negative). Figure 11, Figure 12 and Figure 13 show, using the F1 score as a measure to penalize both false positives and false negatives, how different “resistance” criteria affect model performance when using all three biochemical windows together and increasing principal components. Performance mimics overall accuracy and sees progressively smaller increases as more components are used. Regardless of antibiotic-specific behavior, it should be noted that this application, which is much closer to the overarching goal of simply detecting what is defined as resistance, yielded a model with an impressive ability to distinguish between sample groups. For both Azy and Trim samples, the model approached perfect binary classification performance when it was distinguishing between 0 h and all other groups, i.e., between no antibiotic exposure and any exposure at all. Performance was worse when placing this decision boundary between 24 and 72 h of exposure, and even worse when only counting 120 h of exposure as resistant. This indicates that the samples become more similar to each other the longer they are able to develop resistance in the presence of antibiotics. This has been observed in our prior analysis [9]. Interestingly, the Oxa samples diverge from this pattern as soon as more than one principal component is used, with the delineation between 120 h samples and all other samples producing the best performance. This is likely related to the behavior observed in the prior analysis of MIC, where Oxa samples seemed to increase in resistance and then decline to a value close to the initial state around 120 h [9,11,12,17]. Table A4, Table A5 and Table A6 (In Appendix A) contain the values from these plots of F1 scores.

The temporal average FTIR spectral data presented in Figure 14, Figure 15 and Figure 16 provide information on the general spectral changes in the biochemical windows with prolonged exposure to the three studied antibiotics. In Figure 14, the samples treated with azithromycin, which inhibits the protein synthesis of the bacteria, have a similar pattern in all three biochemical groups [18]. The average FTIR spectral profile peaks after 24 h and decreases after 72 and 120 h. It is worth noting that all three graphs start at different average values and decrease to different lower points. It is observed that values in both the carbohydrate and fatty acid windows decrease below the initial point, but the protein window values do not. In Figure 15, the samples treated with oxacillin, which targets bacterial cell wall synthesis, have a similar pattern across the fatty acid and protein windows, but a very different behavior for carbohydrates [19]. The values progressively increase until reaching a peak at 72 h and then falling for both fatty acids and proteins. This final fall is the largest for fatty acids. The spectral profile for carbohydrates, in contrast, decreases from 0 to 24 h and then progressively increases up to 120 h. In Figure 16, the samples treated with trimethoprim/sulfamethoxazole, which inhibits folate synthesis and indirectly disrupts nucleotide and protein biosynthesis, have a similar pattern for carbohydrates and fatty acids, but a different pattern observed for the protein window [20]. The FTIR spectral profile peaks at 24 h and is lower for the two subsequent points. This fall from 24 to 72 h is drastic for carbohydrates and fatty acids, and it is followed by a small increase to the value at 120 h. This fall, however, is much smaller for proteins and is followed by an additional decline to the value at 120 h.

## 4. Materials and Methods

### 4.1. Sample Preparation and FTIR Spectra Acquisition

*Staphylococcus aureus* (NIST 0023) was cultured and subjected to resistance induction via repeated exposure to 1.5x MIC concentrations of azithromycin (Azy), trimethoprim/sulfamethoxazole (Trim), and oxacillin (Oxa), with MIC monitored at 12 h intervals as previously described [9]. FTIR spectra were acquired from antibiotic-treated samples using Attenuated Total Reflection (ATR) on the Agilent Cary 630 FTIR Spectrometer^®^ instrument (Agilent Technologies, Billerica, MA, USA) n the wavelength range of 650–4000 cm^−1^, 4 cm^−1^ resolution, 250 scans per sample, following the protocol of Soares et al. [10,12]. A total of 1100 spectra were collected.

### 4.2. Data Preparation and Machine Learning Application

(a) Data Processing and Principal Component Analysis (PCA)

Spectral data were processed as previously described [9]. Briefly, raw FTIR spectra were analyzed using Python (3.12.3) [21,22,23,24] and MATLAB (R2021b) [14], employing second derivative analysis, Savitzky–Golay smoothing, and min–max normalization. Spectral regions corresponding to carbohydrates (950–1200 cm^−1^), proteins (1500–1800 cm^−1^), and fatty acids (2800–3100 cm^−1^) were then manually isolated. PCA was applied to each antibiotic and biochemical window using Scikit-learn in Python and R (version 4.2.3) to reduce dimensionality and visualize variation related to antibiotic exposure [21,23,25,26,27,28,29,30,31].

(b) Random Forest Model and Feature Evaluation

A single approach was developed to, at the same time, analyze sample classification and how it is affected by both changes in the number of principal components used and resistance criteria. The general approach was to use a machine learning algorithm to classify these samples using principal components as features. A Random Forest model with 1000 estimators was trained on a random subset containing 80% of the data and then used to predict the class of the other 20%. This was only carried out within each antibiotic dataset so that the focus was time dependence. Every test was carried out 30 times to produce average and standard deviation values.

Given that the overarching goal of this kind of analysis is to be able to quickly classify a sample as “resistant” or “non-resistant” (i.e., susceptible) to specific antibiotics, it was important to test the ability to differentiate samples at different points along the resistance development pathway. For this reason, for the calculation of the F1 score (a balanced way of looking at both precision and recall), different points were chosen to be the “cutoff” for non-resistance such that the classification ability could be compared. For example, for the same classification of a set of data into the different time labels, “resistant” samples would first be only 120 h samples, then 72 and 120 h samples, and then 24, 72, and 120 h samples. The F1 scores for this binary labeling system could then be compared across the tests.

In previous studies it was discovered that there is a significant amount of variance accounted for by the principal components beyond the first two for the protein window. The previously described F1 score was charted as the number of components used increased, but the additional measure of accuracy, or the ability to correctly classify each sample as 0, 24, 72, or 120 h, was also analyzed against the number of components used. This was carried out to observe how overall model performance changed with increasing component use without considering the introduction of noise.

**Figure 17:**
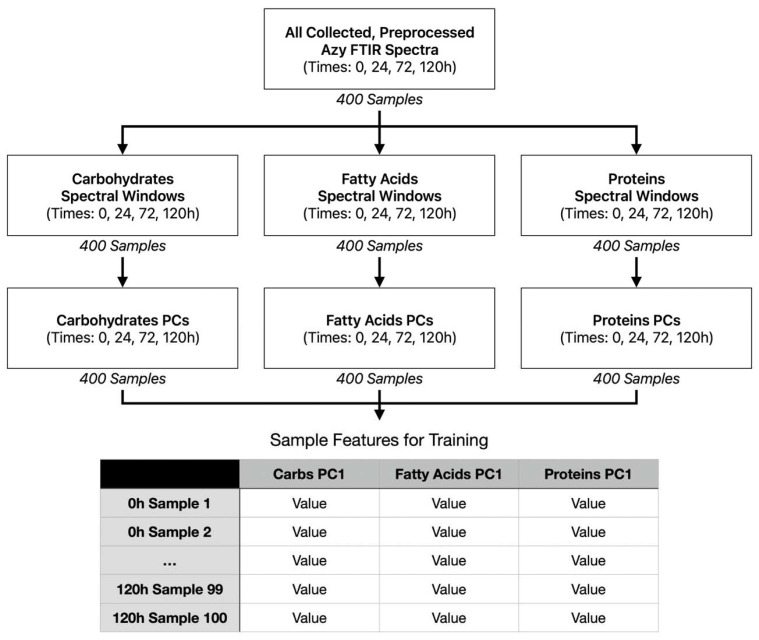
Flowchart of example feature creation for model training (Azy samples, all windows included 1 PC per window).

The overall accuracy calculations were carried out with each individual biochemical window (proteins, fatty acids, and carbohydrates), as well as with all three together, utilizing separate PCA calculations (see Figure 17). The F1 score calculations were only carried out with all three biochemical windows contributing features.

## 5. Conclusions

Collectively, the average spectral data demonstrate the dynamic and time-dependent nature of the bacteria’s biochemical responses to antibiotic exposure. Each antibiotic produced a different time-dependent spectral behavior in the bacteria, with variation across biochemical windows. This indicates the complex evolution of resistance in the treated samples and the benefit of using all biochemical windows in analyses for a better understanding of resistance evolution. The observation of improved model accuracy when using all three windows together (rather than individually) coupled with these unique window behaviors justifies the continued use of all three biomolecule windows when developing features for a prediction model for the sake of more effective resistance identification.

A prediction model performance analysis with increasing numbers of principal components showed diminishing returns with each additional component, with the most significant gains introduced by the first few components. Importantly, the role of noise specific to the collection environment of each time group in the improvements provided by each additional component is unknown and likely nontrivial. The application, therefore, of more complex principal component retention methods alongside the classification of new, independent data would greatly aid feature selection decisions as they apply to each individual window. In general, these results also showed that time-group classification is most effective when trying to determine which samples have had any antibiotic exposure compared to none, rather than separating samples of different exposure times. The latter, however, is still reasonably possible. This impressive binary delineation performance, achieved with a large volume of data, indicates a very promising contribution to the pursuit of a safe, rapid, and accurate method for identifying antibiotic resistance that can be applied to microorganisms and antibiotics not studied here.

While this analysis can offer a helpful glimpse into the potential for antibiotic resistance detection, a more comprehensive understanding would be gained from attempting to classify a larger set of additional samples collected independently using our current data for training. This would allow for a focused effort on feature selection and a better understanding of predictive limitations, particularly if our data introduces environment-specific noise that could inflate performance metrics. It would also be beneficial to analyze the extent to which a model can predict the specific antibiotic to which a sample has developed resistance.

## Data Availability

Data is unavailable due to privacy or ethical restrictions.

## Supplementary Materials

The following supporting information can be downloaded at: https://www.mdpi.com/article/10.3390/antibiotics14080831/s1. Figure S1. (**a**–**d**) One hundred FTIR absorbance spectra into the carbohydrates window obtained for *S. aureus* samples with antibiotic resistance induced to Azy at 0 h, 24 h, 72 h, and 120 h. (**e**–**h**) FTIR spectra region after normalized process done; Figure S2. (**a**–**d**) One hundred FTIR absorbance spectra into the fatty acids window obtained for *S. aureus* samples with antibiotic resistance induced to Azy at 0 h, 24 h, 72 h, and 120 h. (**e**–**h**) FTIR spectra region after normalized process done; Figure S3. (**a**–**d**) One hundred FTIR absorbance spectra into the protein window obtained for *S. aureus* samples with antibiotic resistance induced to Azy at 0 h, 24 h, 72 h, and 120 h. (**e**–**h**) FTIR spectra region after normalized process done; Figure S4. (**a**–**d**) One hundred FTIR absorbance spectra into the carbohydrates window obtained for *S. aureus* samples with antibiotic resistance induced to Oxa at 0 h, 24 h, 72 h, and 120 h. (**e**–**h**) FTIR spectra region after normalized process done; Figure S5. (**a**–**d**) One hundred FTIR absorbance spectra into the fatty acids window obtained for *S. aureus* samples with antibiotic resistance induced to Oxa at 0 h, 24 h, 72 h, and 120 h. (**e**–**h**) FTIR spectra region after normalized process done; Figure S6. (**a**–**d**) One hundred FTIR absorbance spectra into the protein window obtained for *S. aureus* samples with antibiotic resistance induced to Oxa at 0 h, 24 h, 72 h, and 120 h. (**e**–**h**) FTIR spectra region after normalized process done; Figure S7. (**a**–**d**) One hundred FTIR absorbance spectra into the carbohydrates window obtained for *S. aureus* samples with antibiotic resistance induced to Trim at 0 h, 24 h, 72 h, and 120 h. (**e**–**h**) FTIR spectra region after normalized process done; Figure S8. (**a**–**d**) One hundred FTIR absorbance spectra into the fatty acids window obtained for *S. aureus* samples with antibiotic resistance induced to Trim at 0 h, 24 h, 72 h, and 120 h. (**e**–**h**) FTIR spectra region after normalized process done; Figure S9. (**a**–**d**) One hundred FTIR absorbance spectra into the protein window obtained for *S. aureus* samples with antibiotic resistance induced to Trim at 0 h, 24 h, 72 h, and 120 h. (**e**–**h**) FTIR spectra region after normalized process done.

## Appendix A

Table A1, Table A2 and Table A3 show the results of calculating overall accuracy for the prediction model’s time-group classification. This was found while increasing the number of used principal components, utilizing features from each of the three biochemical windows separately and together. Results for all three tested antibiotics are shown.

**Table A1:** Classification accuracy for increasing principal components for different features (individual biomolecules vs. all together) (Azy).

PCs Per Window	Overall Accuracy (Mean ± Std.)			
	All Windows	Proteins	Fatty Acids	Carbohydrates
1	30 ± 4.8%	24.2 ± 4.2%	26.3 ± 4.3%	23 ± 3.1%
2	50.4 ± 7.5%	32.1 ± 5.8%	38.2 ± 5.2%	31 ± 4.3%
3	68.3 ± 4.1%	37.6 ± 4.0%	54 ± 6.1%	53.9 ± 4.2%
4	77.8 ± 4.5%	43.7 ± 4.0%	65.2 ± 6.7%	62.5 ± 4.2%
5	82 ± 4.7%	46.5 ± 4.8%	69.2 ± 5.2%	67 ± 4.5%
6	83.8 ± 3.5%	51.3 ± 4.5%	72.4 ± 4.3%	71.2 ± 4.2%
7	84.9 ± 3.3%	52.7 ± 4.3%	75 ± 4.7%	75.4 ± 3.5%

**Table A2:** Classification accuracy for increasing principal components for different features (individual biomolecules vs. all together) (Oxa).

PCs Per Window	Overall Accuracy (Mean ± Std.)			
	All Windows	Proteins	Fatty Acids	Carbohydrates
1	32.2 ± 6.1%	26.5 ± 5.0%	26.8 ± 4.5%	26.2 ± 3.9%
2	67.9 ± 4.4%	27.3 ± 3.8%	37.8 ± 3.6%	63.3 ± 3.7%
3	81.2 ± 3.4%	35.2 ± 3.5%	48.7 ± 5.8%	76.2 ± 4.3%
4	84.4 ± 3.6%	41.5 ± 4.8%	56.7 ± 5.4%	81.1 ± 3.7%
5	88.5 ± 3.6%	43.8 ± 4.7%	61.8 ± 5.6%	87.8 ± 3.2%
6	96.1 ± 2.3%	46.3 ± 5.0%	65.8 ± 5.6%	93.1 ± 3.4%
7	96.4 ± 2.1%	51.1 ± 6.0%	70.7 ± 5.0%	93.9 ± 3.2%

**Table A3:** Classification accuracy for increasing principal components for different features (individual biomolecules vs. all together) (Trim).

PCs Per Window	Overall Accuracy (Mean ± Std.)			
	All Windows	Proteins	Fatty Acids	Carbohydrates
1	27.5 ± 5.4%	26.1 ± 4.7%	28.1 ± 4.0%	21.3 ± 4.0%
2	43.8 ± 6.0%	25.5 ± 5.2%	35.0 ± 4.7%	34.3 ± 4.6%
3	59.8 ± 5.5%	28.6 ± 5.1%	42.1 ± 4.5%	49.3 ± 5.5%
4	69.8 ± 5.6%	36.7 ± 4.9%	50.0 ± 3.8%	63.8 ± 4.2%
5	75.2 ± 4.8%	40.3 ± 5.7%	56.3 ± 5.1%	68.1 ± 5.1%
6	79.3 ± 4.2%	41.8 ± 6.2%	60.6 ± 4.6%	73.6 ± 4.3%
7	82.6 ± 4.5%	42.7 ± 4.8%	65.5 ± 4.3%	76.7 ± 4.7%

Table A4, Table A5 and Table A6 show the results of calculating the F1 score for each “positive” resistance criteria, or different sample groups considered resistant, while increasing the number of used principal components. These tests utilized features (principal components) from all three biochemical windows and were carried out with all three tested antibiotics.

**Table A4:** F1 for increasing principal components (Azy).

PCs Per Window	F1 Score (Mean ± Std.)		
	Positive: 120 h	Positive: 72, 120 h	Positive: 24, 72, 120 h
1	0.313 ± 0.090	0.556 ± 0.051	0.789 ± 0.036
2	0.431 ± 0.102	0.666 ± 0.073	0.892 ± 0.032
3	0.543 ± 0.074	0.776 ± 0.042	0.953 ± 0.022
4	0.655 ± 0.083	0.835 ± 0.035	0.987 ± 0.011
5	0.744 ± 0.079	0.859 ± 0.040	0.994 ± 0.007
6	0.781 ± 0.063	0.866 ± 0.044	0.994 ± 0.008
7	0.804 ± 0.048	0.873 ± 0.038	0.993 ± 0.008

**Table A5:** F1 for increasing principal components (Oxa).

PCs Per Window	F1 Score (Mean ± Std.)		
	Positive: 120 h	Positive: 72, 120 h	Positive: 24, 72, 120 h
1	0.433 ± 0.100	0.534 ± 0.068	0.768 ± 0.042
2	0.904 ± 0.040	0.751 ± 0.044	0.860 ± 0.030
3	0.943 ± 0.028	0.874 ± 0.029	0.933 ± 0.018
4	0.947 ± 0.036	0.886 ± 0.036	0.939 ± 0.018
5	0.967 ± 0.028	0.905 ± 0.027	0.943 ± 0.017
6	0.989 ± 0.017	0.973 ± 0.020	0.981 ± 0.013
7	0.993 ± 0.015	0.976 ± 0.018	0.980 ± 0.014

**Table A6:** F1 for increasing principal components (Trim).

PCs Per Window	F1 Score (Mean ± Std.)		
	Positive: 120 h	Positive: 72, 120 h	Positive: 24, 72, 120 h
1	0.259 ± 0.073	0.485 ± 0.078	0.755 ± 0.046
2	0.412 ± 0.103	0.650 ± 0.058	0.788 ± 0.034
3	0.645 ± 0.081	0.746 ± 0.049	0.852 ± 0.030
4	0.703 ± 0.065	0.812 ± 0.053	0.888 ± 0.026
5	0.752 ± 0.052	0.836 ± 0.047	0.911 ± 0.026
6	0.780 ± 0.063	0.852 ± 0.040	0.929 ± 0.022
7	0.807 ± 0.059	0.874 ± 0.044	0.936 ± 0.022
