# Supplementary material for: FTIR-Derived Feature Insights for Predicting Time-Dependent Antibiotic Resistance Progression
Source: Antibiotics (Basel). 2025 Aug 15;14(8):831. doi: 10.3390/antibiotics14080831 (PMC12382883; doi:10.3390/antibiotics14080831)
Supplement: Supplementary file 1 [file antibiotics-14-00831-s001.zip › antibiotics-3720660-supplementary.pdf]

antibiotics

MDPI

Article supplementary material

FTIR-Derived Feature Insights for Predicting Time-Dependent Antibiotic Resistance Progression

Mitchell Bonner [1,*], Claudia P. Barrera Patiño [1,2,*], Andrew Ramos Borsatto [1], Jennifer M. Soares [1,2], Kate C. Blanco [2] and Vanderlei S. Bagnato [1,2]

[1] Biomedical Engineering, Texas A&M University, 400 Bizzell St, College Station, TX 77843, United States of America; andrewrborsatto@tamu.edu (A.R.B.); jennifer.soares@usp.br (J.M.S.); vander@ifsc.usp.br or bagnatovs@tamu.edu (V.S.B.)
[2] Sao Carlos Institute of Physics, University of Sao Paulo, IFSC – USP, 13566-590, Sao Carlos, SP, Brazil; kateblanco@ifsc.usp.br (K.C.B.)
* Correspondence: mitchellabonner@tamu.edu, cpbarrerapatino@tamu.edu or cpbarrerap@ifsc.usp.br

FTIR spectra of *S. aureus* at 0h, 24h, 72h, and 120h for the antibiotics Azy, Oxa, and Trim

In this section we supply the FTIR spectra of *S. aureus* bacteria inside the carbohydrates, fatty acids and protein windows at 0h, 24h, 72h, and 120h for the antibiotics: azithromycin (Azy), oxacillin (Oxa), and trimethoprim/sulfamethoxazole (Trim). The last join with the results obtained from normalization by maximum value of each one of the one hundred FTIR spectra from the samples studied in vary time for each one of three different antibiotics.

The process implemented is developed in the next way: the samples were acquired following the procedure report by Soares *et al.* in [1,2] with resistance-induced strains protocol by Soares *et al.* in [3]. In addition, the data processing was developed follows the steps of the protocol of Naumann *et at.* [4] implemented in owner code in MATLAB R2021b [5]. The data analyses of the FTIR absorption spectrum of *S. aureus* bacteria were processed according to the next steps [6]:

(i) FTIR absorption spectra acquisition one by one [1].

(ii) Second derivative calculation for each spectrum individually. Each time group is conforming by one hundred FTIR absorption spectra in to each antibiotic implemented into the study [4].
The last is done by mean of the implementation of the second-order difference of dataset. That's mean that each point into the spectrum data set is associated to one vector (λ_1, I_1). It corresponds to one array forming by the wavelength value and its correspondent FTIR absorption intensity record value. Then, each vector is processed to compute with the second-order difference. This method lets also calculates differences between adjacent elements. The entire calculation process was developed with default functions available in MATLAB R2021b [5].

(iii) Normalization by maximum value of FTIR absorption intensity [4], process done in each one spectrum individually.

(iv) Extract the window interval group, it is conforming the array of one hundred FTIR absorption spectra intensity with the same wavelength values [4].

Figures S1-S9 show the FTIR spectra region inside the carbohydrates, fatty acids and protein windows, and the normalized FTIR spectra results of *S. aureus* bacteria for each one of the biochemical group intervals studied here at 0h, 24h, 72h, and 120h for the antibiotics Azy, Oxa, and Trim.

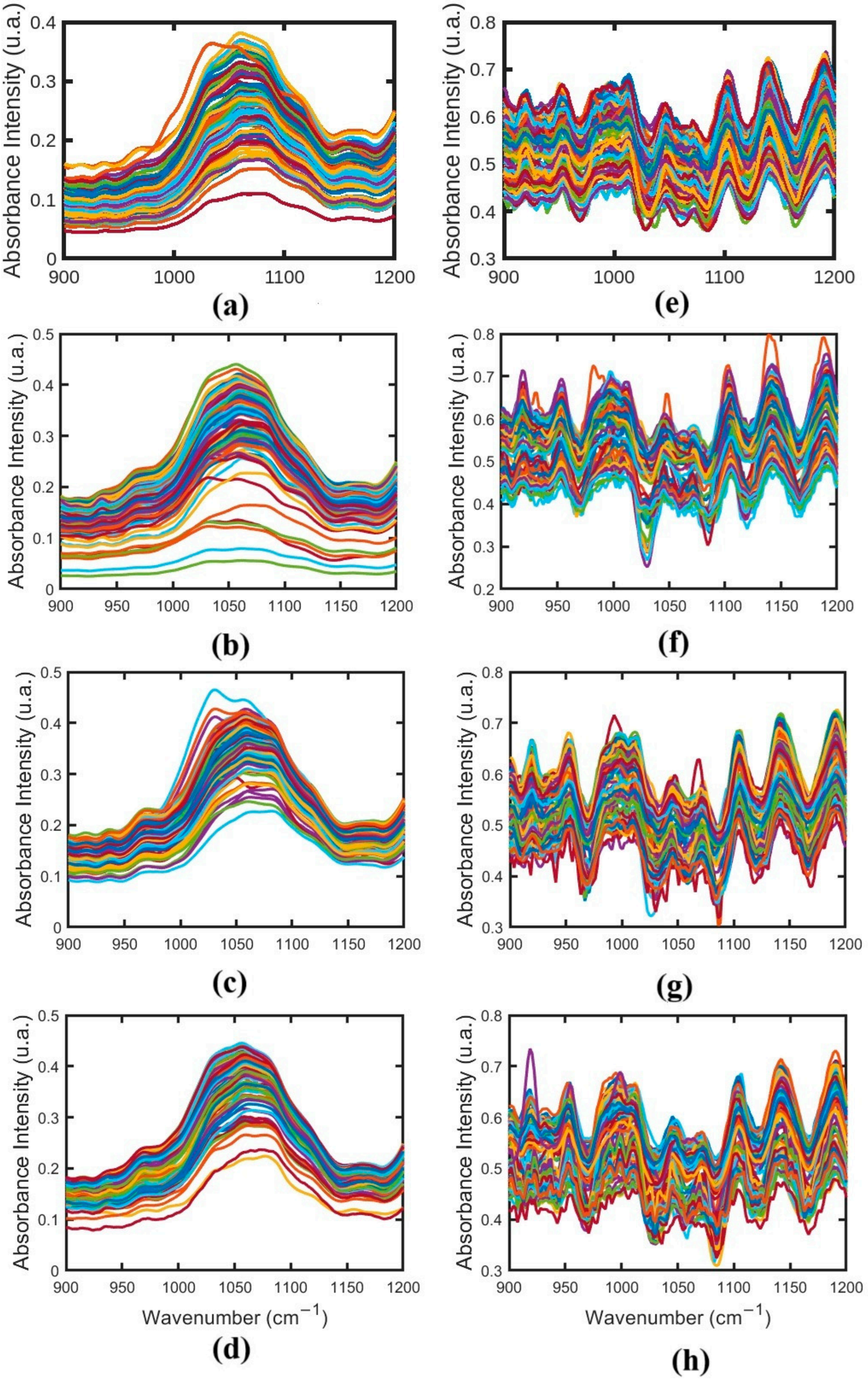

Figure S1. (a-d) One hundred FTIR absorbance spectra into the carbohydrates window obtained for *S. aureus* samples with antibiotic resistance induced to Azy at 0h, 24h, 72h, and 120h. (e-h) FTIR spectra region after normalized process done.

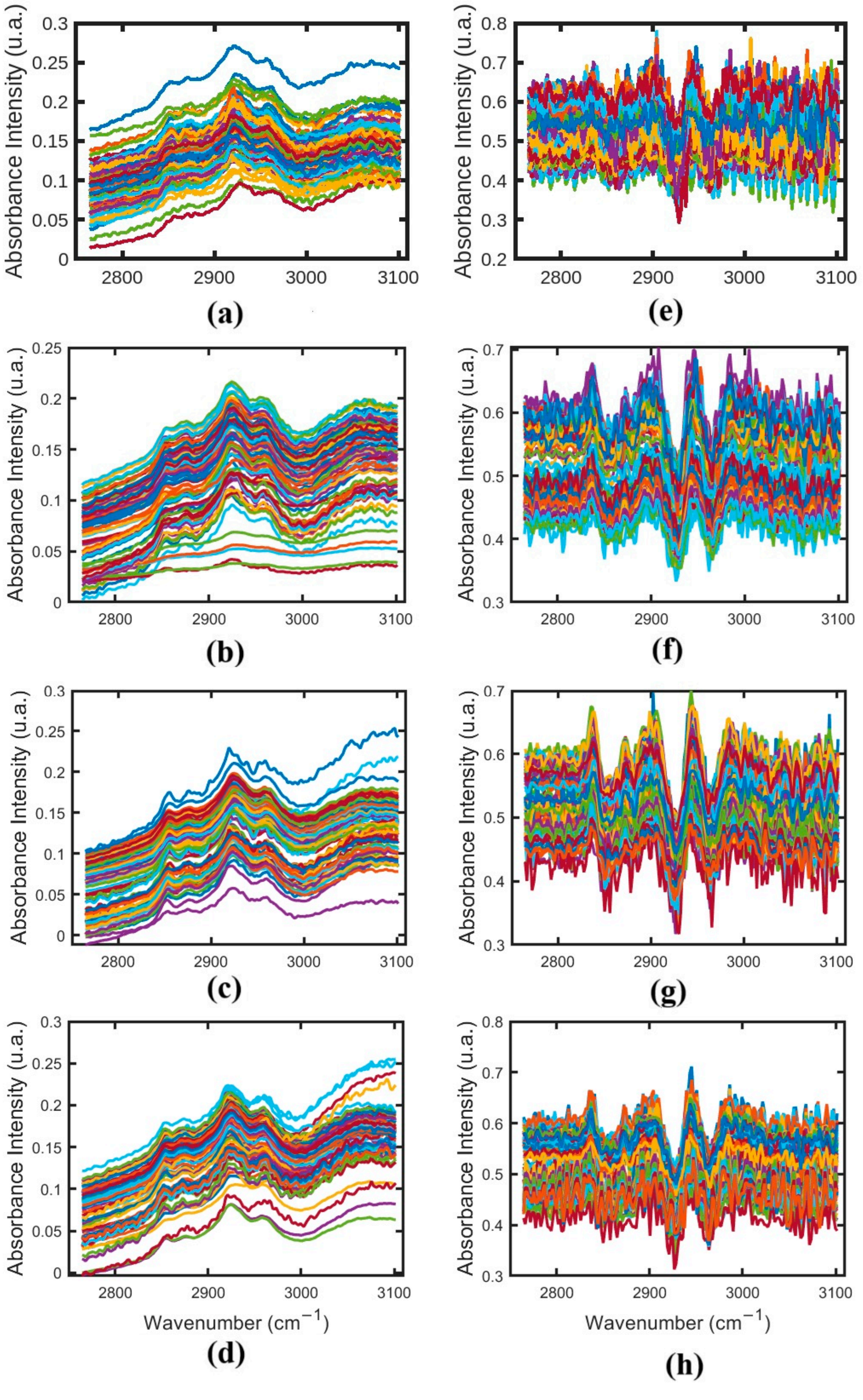

Figure S2. (a-d) One hundred FTIR absorbance spectra into the fatty acids window obtained for *S. aureus* samples with antibiotic resistance induced to Azy at 0h, 24h, 72h, and 120h. (e-h) FTIR spectra region after normalized process done.

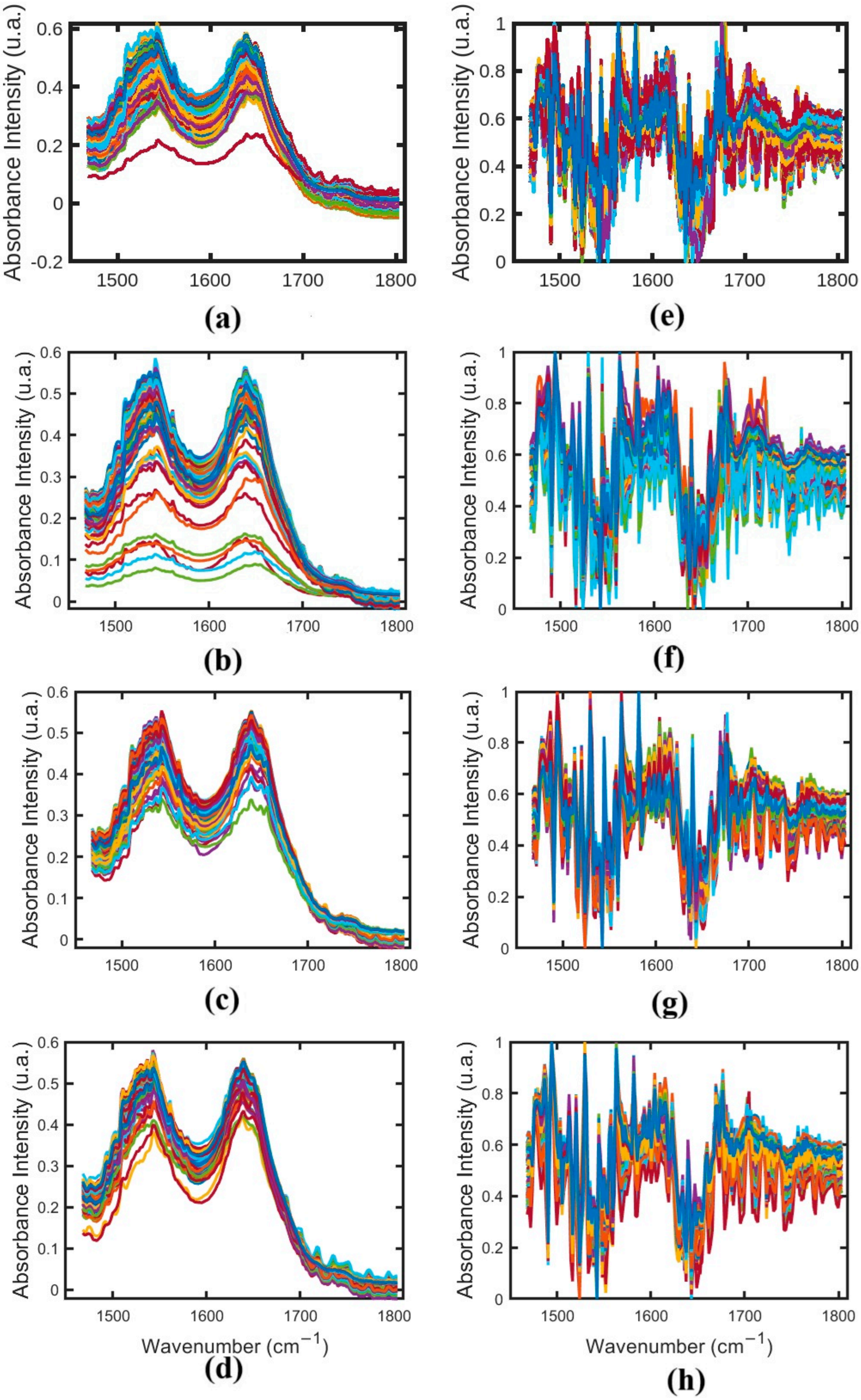

Figure S3. (a-d) One hundred FTIR absorbance spectra into the protein window obtained for *S. aureus* samples with antibiotic resistance induced to Azy at 0h, 24h, 72h, and 120h. (e-h) FTIR spectra region after normalized process done.

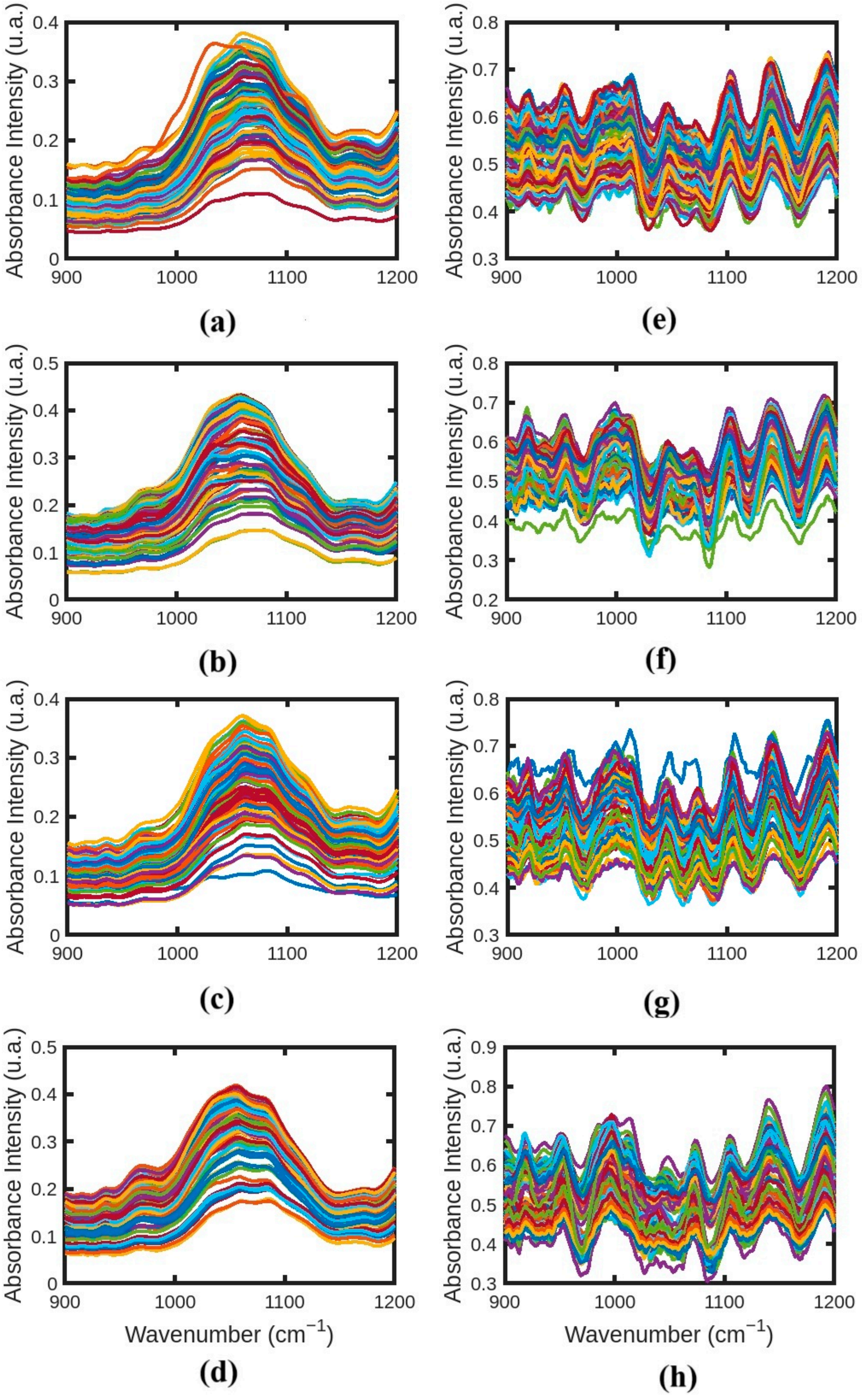

Figure S4. (a-d) One hundred FTIR absorbance spectra into the carbohydrates window obtained for *S. aureus* samples with antibiotic resistance induced to Oxa at 0h, 24h, 72h, and 120h. (e-h) FTIR spectra region after normalized process done.

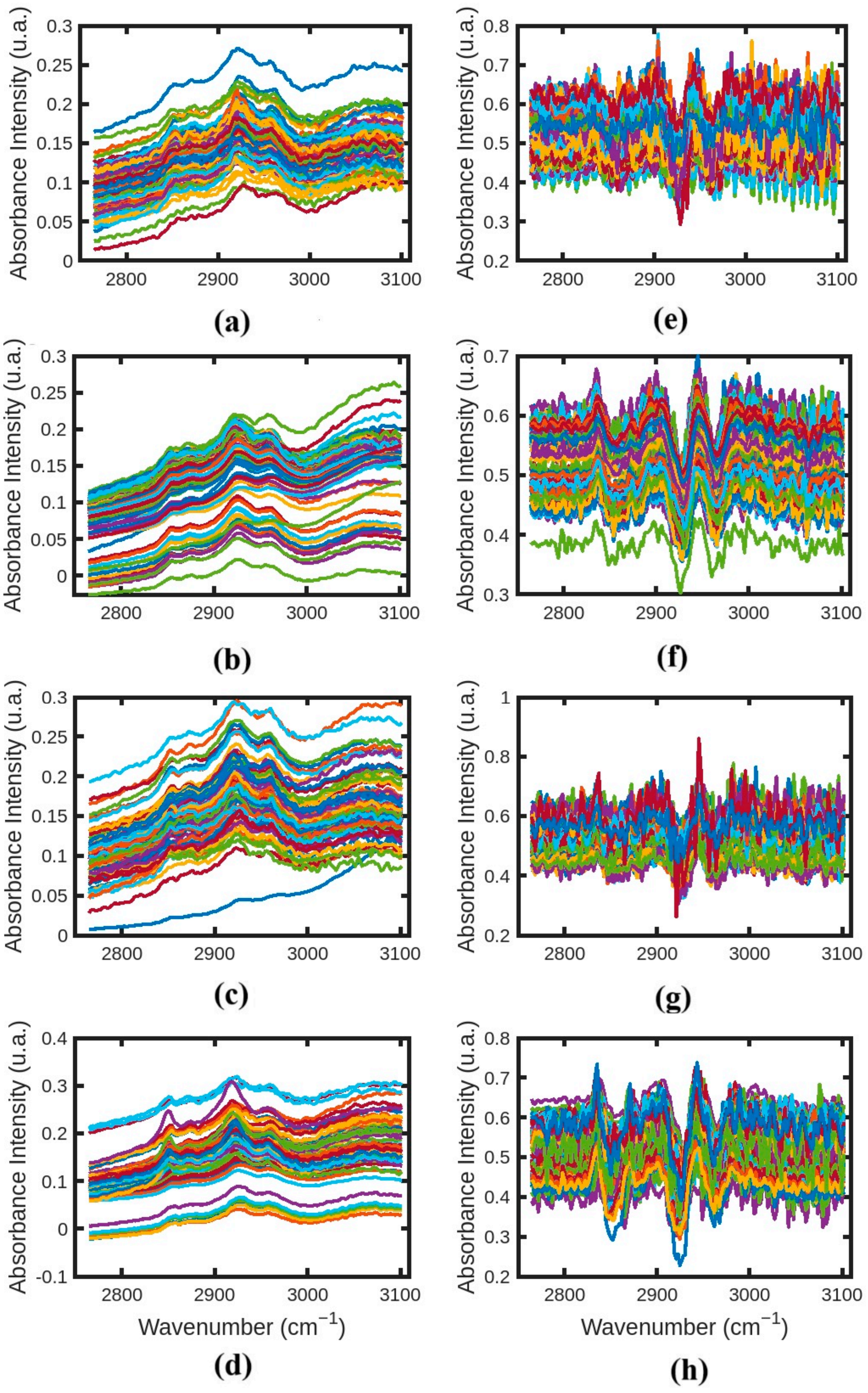

Figure S5. (a-d) One hundred FTIR absorbance spectra into the fatty acids window obtained for *S. aureus* samples with antibiotic resistance induced to Oxa at 0h, 24h, 72h, and 120h. (e-h) FTIR spectra region after normalized process done.

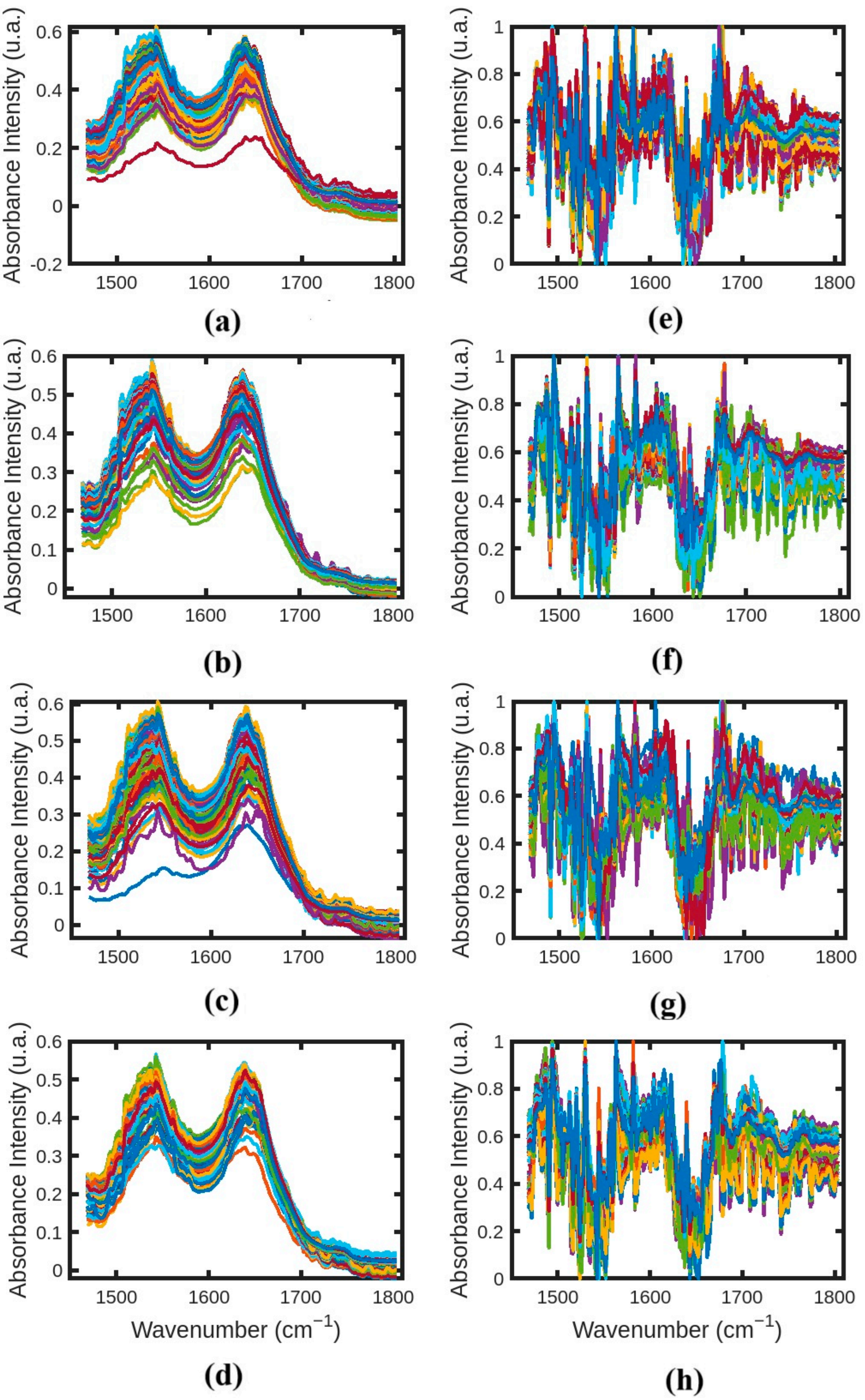

Figure S6. (a-d) One hundred FTIR absorbance spectra into the protein window obtained for *S. aureus* samples with antibiotic resistance induced to Oxa at 0h, 24h, 72h, and 120h. (e-h) FTIR spectra region after normalized process done.

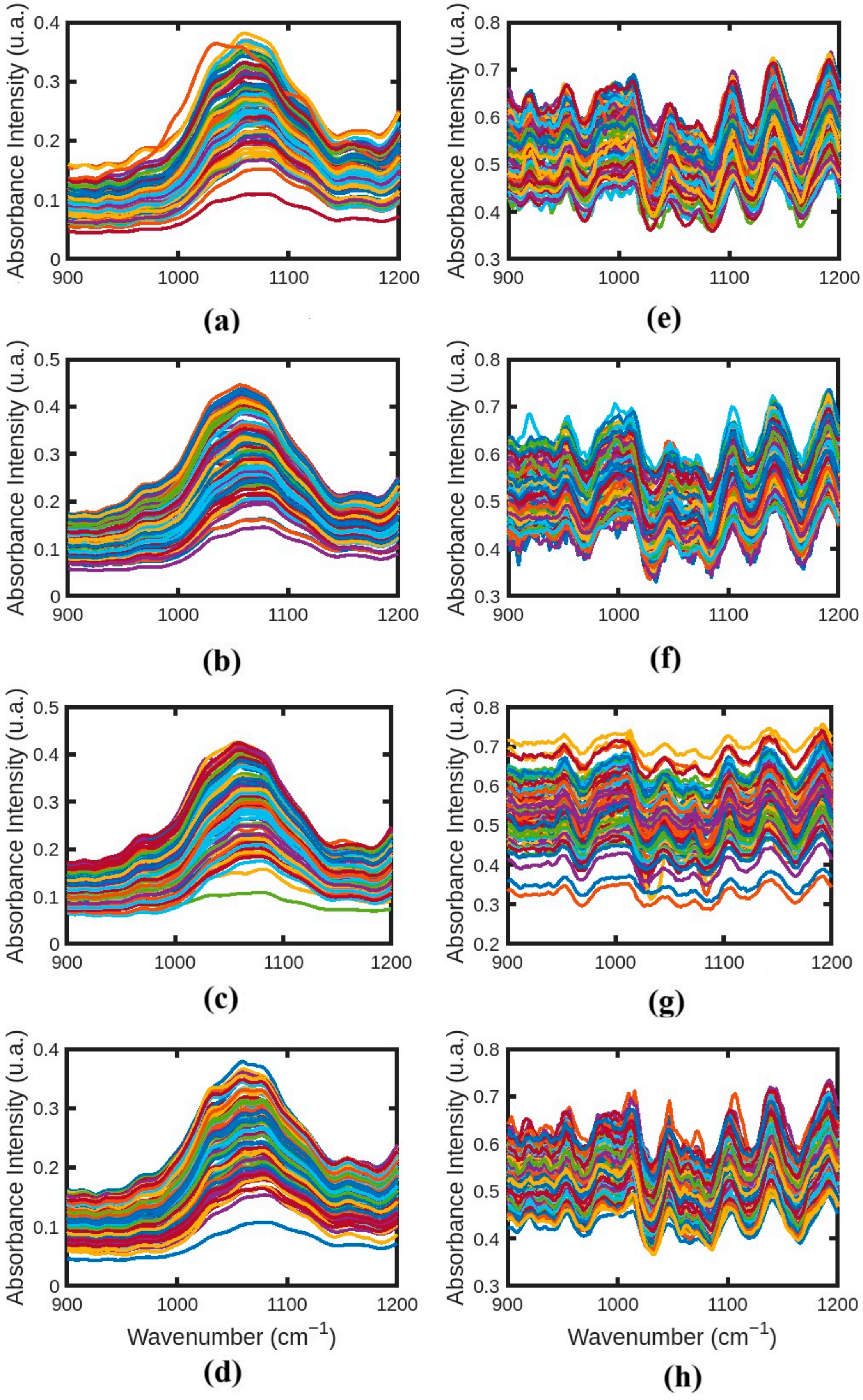

Figure S7. (a-d) One hundred FTIR absorbance spectra into the carbohydrates window obtained for *S. aureus* samples with antibiotic resistance induced to Trim at 0h, 24h, 72h, and 120h. (e-h) FTIR spectra region after normalized process done.

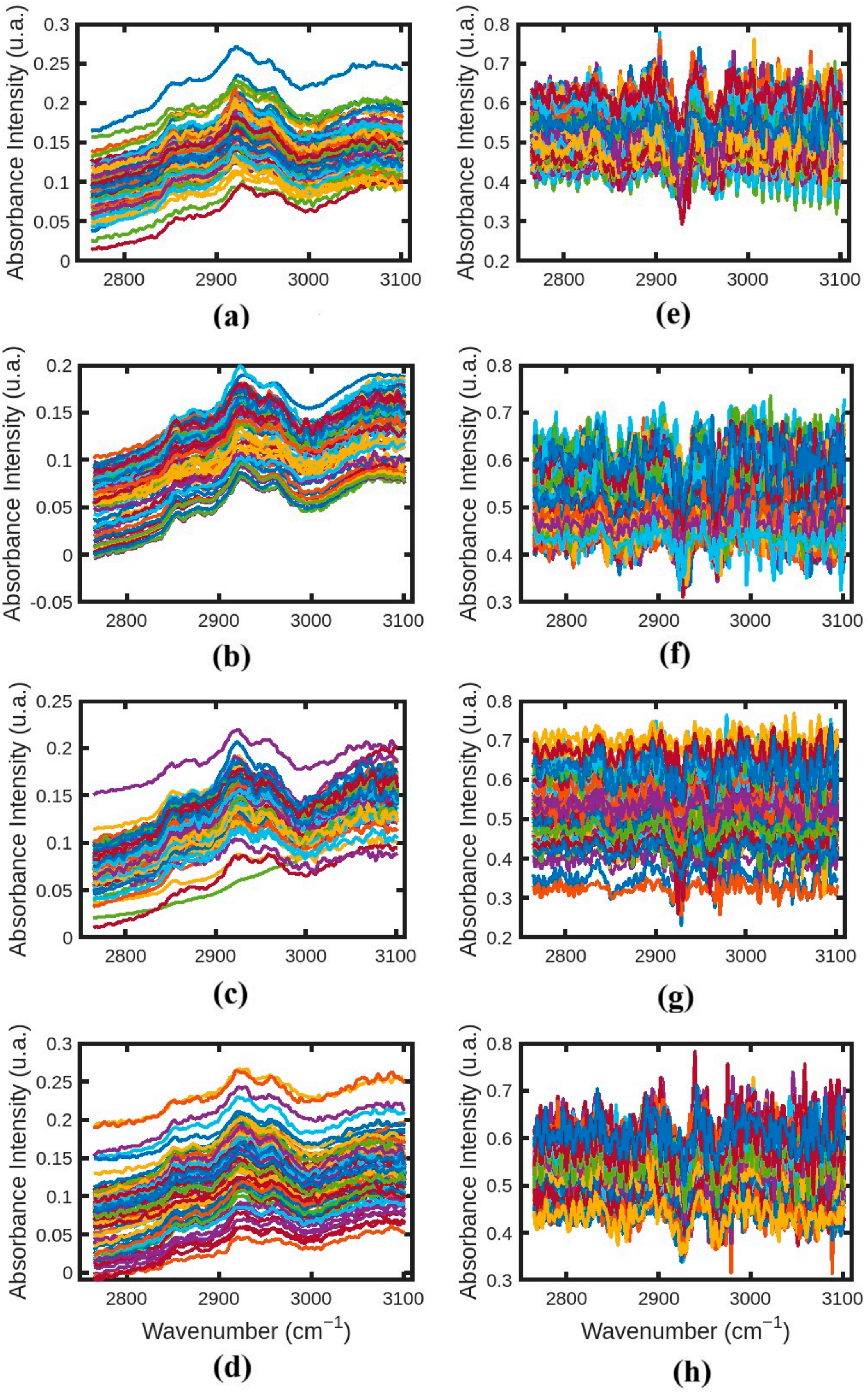

Figure S8. (a-d) One hundred FTIR absorbance spectra into the fatty acids window obtained for *S. aureus* samples with antibiotic resistance induced to Trim at 0h, 24h, 72h, and 120h. (e-h) FTIR spectra region after normalized process done.

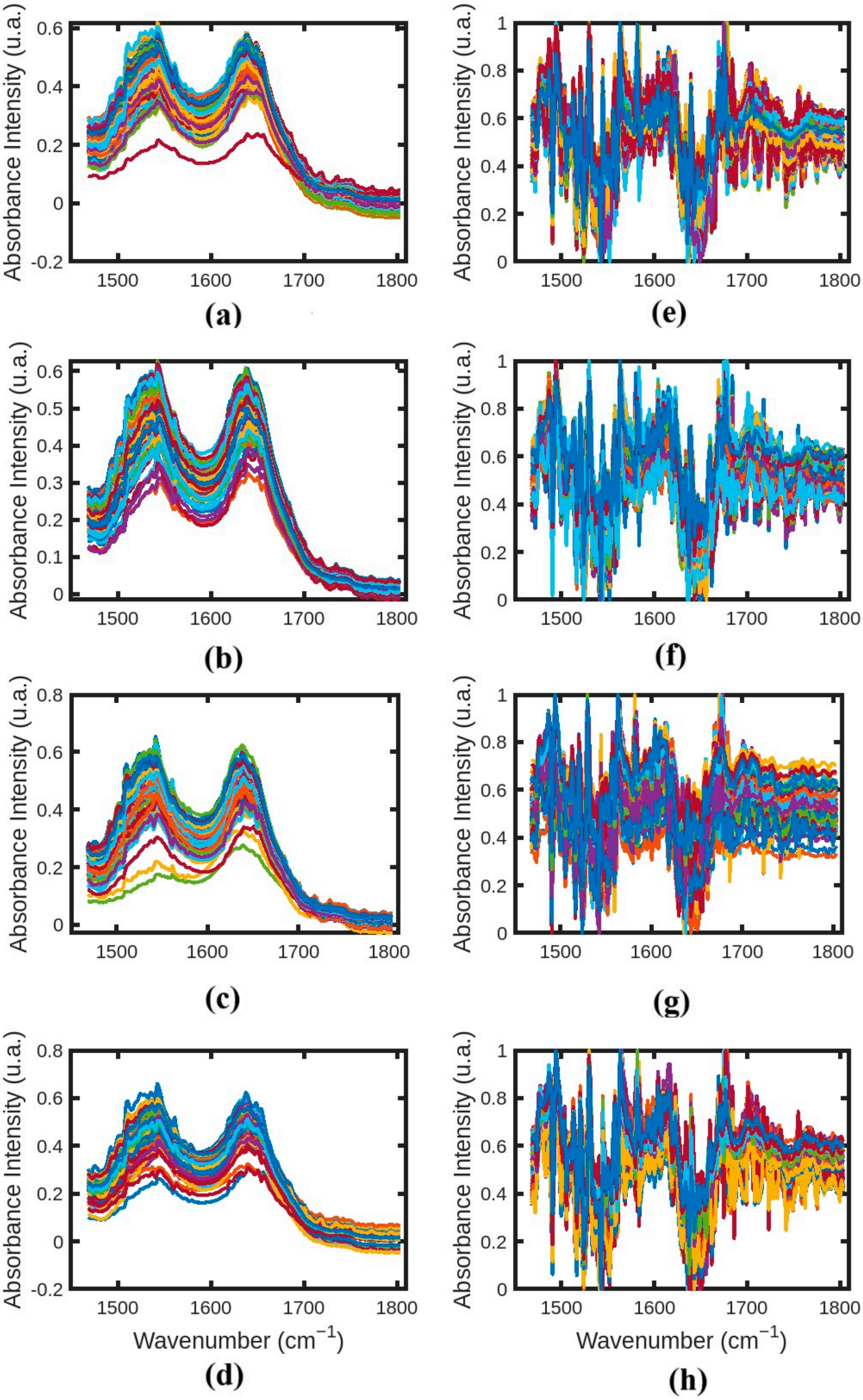

Figure S9. (a-d) One hundred FTIR absorbance spectra into the protein window obtained for *S. aureus* samples with antibiotic resistance induced to Trim at 0h, 24h, 72h, and 120h. (e-h) FTIR spectra region after normalized process done.

References

1. Soares, J.M.; Guimarães, F.E.G.; Yakovlev, V. V.; Bagnato, V.S.; Blanco, K.C. Physicochemical Mechanisms of Bacterial Response in the Photodynamic Potentiation of Antibiotic Effects. *Sci. Rep.* **2022**, *12*, 21146, doi:10.1038/s41598-022-25546-y.
2. Willis, J.A.; Cheburkanov, V.; Chen, S.; Soares, J.M.; Kassab, G.; Blanco, K.C.; Bagnato, V.S.; de Figueiredo, P.; Yakovlev, V. V. Breaking down Antibiotic Resistance in Methicillin-Resistant *Staphylococcus Aureus* : Combining Antimicrobial Photodynamic and Antibiotic Treatments. *Proc. Natl. Acad. Sci.* **2022**, *119*, doi:10.1073/pnas.2208378119.
3. Soares, J.M.; Yakovlev, V. V.; Blanco, K.C.; Bagnato, V.S. Recovering the Susceptibility of Antibiotic-Resistant Bacteria Using Photooxidative Damage. *Proc. Natl. Acad. Sci.* **2023**, *120*, doi:10.1073/pnas.2311667120.
4. Naumann, D. Infrared Spectroscopy in Microbiology. *Encycl. Anal. Chem.* **2000**, *102*, 131.
5. The MathWorks Inc. MATLAB R2021b. *https://www.mathworks.com* **2021**.
6. Barrera-Patiño, C.P.; Soares, J.M.; Branco, K.C.; Inada, N.M.; Bagnato, V.S. Spectroscopic Identification of Bacteria Resistance to Antibiotics by Means of Absorption of Specific Biochemical Groups and Special Machine Learning Algorithm. *Antibiotics* **2023**, *12*, 1502, doi:10.3390/antibiotics12101502.
